# Does the Forensic Filler-Control Method Reduce Examiner Overconfidence? An Experimental Investigation Using Mock Fingerprint Examiners

**DOI:** 10.3390/bs15091191

**Published:** 2025-08-31

**Authors:** Hannah J. Rath, Bethany Rocha, Andrew M. Smith, Laura Smalarz

**Affiliations:** 1 School of Interdisciplinary Forensics, Arizona State University, Glendale, AZ 85306, USA; hrath2@asu.edu (H.J.R.); barocha@utmb.edu (B.R.); laura.smalarz@asu.edu (L.S.); 2 Department of Psychology, Iowa State University, Ames, IA 50011, USA; amsmith@iastate.edu

**Keywords:** forensic science, confidence calibration, filler-control method

## Abstract

Examiner overconfidence is a persistent challenge in the field of forensic science, where testimony overstating the validity of forensic techniques has contributed to numerous wrongful convictions. Scholars have proposed a new method for reducing examiner overconfidence (i.e., subjective confidence that exceeds objective accuracy): the forensic filler-control method. The forensic filler-control method, which includes known non-matching “filler” samples alongside the suspect’s sample, is theorized to reduce examiner overconfidence through the provision of immediate error feedback to examiners following match judgments on fillers. We conducted two experiments that failed to yield support for this claim. Among both an undergraduate student sample (Experiment 1) and a forensic science student sample (Experiment 2), the filler-control method was associated with worse calibration (C) and greater overconfidence (O/U) in affirmative match judgments than the standard method. Moreover, the filler-control method produced less accurate non-match judgments, undermining the exonerating value of forensic analysis (i.e., NPV). However, the filler-control method’s ability to draw false positive matches away from innocent-suspect samples and onto fillers produced more reliable incriminating evidence (i.e., PPV) compared to the standard procedure. Our findings suggest that neither the standard procedure nor the filler-control procedure offers a uniformly superior method of conducting forensic analysis. We suggest alternative procedures for enhancing both the inculpatory and exculpatory value of forensic analysis.

## 1. Introduction

Forensic analysis of crime scene evidence such as fingerprints, tool marks, and bullet and cartridge cases typically involves presenting forensic examiners with two samples: one from the crime scene (e.g., a latent fingerprint), and one from the suspect (e.g., the suspect’s fingerprint). The examiners’ task is to determine whether the suspect’s sample matches or does not match the crime scene sample. This method of conducting forensic feature comparison analyses has been involved in thousands of convictions of innocent individuals, making flawed forensic evidence a leading cause of wrongful conviction in the U.S. (National Registry of Exonerations, 2025). In light of this discovery, scholars have suggested an alternative procedure for conducting forensic feature comparison analyses: the filler-control method (Wells et al., 2013), otherwise known as an “evidence lineup” (Kassin et al., 2013; Risinger et al., 2002). Akin to an eyewitness lineup, the filler-control method involves presenting examiners with the crime scene sample and a minimum of two comparison samples: one from the suspect, and at least one “filler” sample that is known not to match the crime scene sample. The examiner then must decide whether any of the comparison samples matches the crime scene sample.

The filler-control method is proposed to have several advantages over the standard forensic analysis method. First, the filler-control method may help prevent forensic confirmation bias—the process whereby an examiner’s beliefs, expectations, motivation, or situational context influence their interpretation of forensic evidence (Kassin et al., 2013). In the standard procedure, examiners’ knowledge of contextual case information (e.g., that the suspect confessed) can lead them to perceive greater similarity between the suspect’s sample and the crime scene sample or lead them to lower their threshold for rendering a match decision (e.g., Cooper & Meterko, 2019; Kukucka & Kassin, 2014). Because examiners using the filler-control method do not know which sample is from the suspect, the filler-control method can reduce the influence of contextual bias on examiners’ judgments (Quigley-McBride & Wells, 2018). Another key benefit of the filler-control method is that it provides a mechanism for exposing errors—namely, match judgments on fillers—that would go undetected in the standard procedure (Wells et al., 2013). The filler-control method thus provides a way to estimate the error rate in actual cases (Wells et al., 2013). Moreover, the filler-control method enables error-rate estimation not only for the forensic technique but for a given laboratory or individual forensic examiner. This feature of the filler-control method underlies the third proposed benefit of the procedure, which is to reduce examiner overconfidence through the provision of error feedback to examiners (Wells et al., 2013). To date, however, no research has tested this purported benefit of the filler-control method. The goal of the current research, therefore, was to test whether the filler-control method reduces examiner overconfidence compared to the standard feature comparison method.

Overconfidence is rampant in the field of forensic science, where “failure to acknowledge uncertainty in findings is common” (National Research Council, 2009, p. 47) and expert witnesses have been criticized for providing testimony that goes “far beyond what the relevant science can justify” (President’s Council of Advisors on Science and Technology, 2016, p. 29). Such high-confidence forensic testimony is persuasive to jurors, who are often more prone to convict when forensic experts downplay or fail to acknowledge the potential for error (Crozier et al., 2020; Garrett & Mitchell, 2013; Koehler, 2011). Indeed, numerous cases of wrongful conviction can be at least partially attributed to testimony from forensic examiners who overstated the validity of their conclusions (Morgan, 2023; Innocence Project, n.d.b). As just one example, Ray Krone received a death sentence for a 1992 murder conviction based largely on forensic bite mark testimony from two examiners. One examiner referred to the bite marks on the murder victim as an “excellent match” to Krone’s teeth and stated that “it was Ray Krone’s teeth” (Garrett & Neufeld, 2009, p. 70). The other examiner testified that Krone’s teeth were a “definite match” (Garrett & Neufeld, 2009, p. 70). After spending more than a decade in prison, Krone was exonerated when DNA evidence conclusively established his innocence (Innocence Project, n.d.a).

Examiner overconfidence may be a result of the illusion of validity, a phenomenon in which people are prone to be highly confident in their judgments despite having poor judgmental accuracy (Kahneman & Tversky, 1973). This phenomenon is pervasive even among trained medical professionals making decisions about patients’ health (Bushyhead & Christensen-Szalanski, 1981). Importantly, the illusion of validity may be the result of incomplete feedback about one’s mistakes (Einhorn & Hogarth, 1978). It is possible that this phenomenon occurs among forensic examiners, who do not receive regular and reliable error feedback about their case judgments (Wells et al., 2013). The standard feature comparison procedure does not provide routine error feedback to forensic examiners because ground truth (i.e., whether the suspect’s sample matches or does not match the crime scene sample) is typically unknown, and false positive errors are often undiscovered until years down the line, if ever. In contrast, the filler-control method provides immediate and regular error feedback to examiners—namely, any time the examiner renders a match judgment on a filler sample. Filler errors are likely to occur after only a few uses of the filler-control method if the technique is unreliable or the examiner is incompetent (Wells et al., 2013). For valid but imperfect forensic techniques, the filler-control method produces an error rate estimate that can help examiners calibrate their confidence with their accuracy. For example, if a hypothetical forensic examiner knew that her error rate for a certain conclusion was 30%, she might learn that she should never be more than 70% confident in that conclusion.

Although no research, to our knowledge, has examined confidence-accuracy calibration in the forensic filler-control method, a variety of studies have demonstrated that the provision of error feedback can reduce overconfidence and improve confidence-accuracy calibration. In an early examination of this phenomenon, (Arkes et al., 1987) asked participants to respond to 35 general knowledge questions by indicating which of two response options was the most likely answer and rating their confidence in the accuracy of their response. After responding to five questions, half of the participants received feedback about the accuracy of their answers to the first five questions; the other half did not. Because average accuracy on the first five questions was around chance (50%), information about the correct answers functioned largely as error feedback. The experimenters then measured participants’ confidence calibration on the remaining 30 questions. Consistent with the idea that providing error feedback improves calibration, participants who received feedback after the first five questions were less overconfident on the remaining 30 questions than were participants who did not receive feedback. More recently, Haddara and Rahnev (2022) investigated whether feedback-induced performance improvements are attributable to automatic sensory processing improvements (i.e., increased perceptual and/or metacognitive sensitivity) or improvements in decision strategy (i.e., changes in response criteria for perceptual and/or metacognitive judgments). They found that trial-by-trial feedback exerts its effects through the latter mechanism, ultimately improving calibration. Most germane to the current research, the authors explained that “when overconfident participants receive feedback about being wrong, this feedback allows them to lower their confidence ratings, thus improving their confidence calibration” (p. 272). Thus, forensic examiners who receive error feedback after rendering mistaken match judgments on filler samples may likewise adjust their confidence ratings to better reflect their accuracy.

However, the provision of error feedback is not the only feature of the filler-control-method that has the potential to affect confidence–accuracy calibration. Procedures that include fillers are inherently more difficult than those that do not (Smith et al., 2020a) because the presence of fillers adds “noise” that makes it more difficult to discriminate between the presence and absence of signal (Macmillan, 2002). This increased task difficulty might work against the calibration-enhancing effects of providing immediate error feedback. According to the hard-easy effect, calibration moves systematically from underconfidence to overconfidence as the difficulty of the task increases (Lichtenstein et al., 1981). In an early study demonstrating the hard-easy effect, Lichtenstein and Fischhoff (1977) asked participants a variety of easy or difficult general knowledge (Experiments 3–5) and psychology (Experiment 4) questions and obtained participants’ probability estimates that their answers were correct. Participants were more overconfident on the hard questions than on the easy questions. This finding has been replicated in a variety of domains, including perceptual judgments (e.g., Baranski & Petrusic, 1994; Petrusic & Baranski, 1997). Thus, the fact that the filler-control method presents examiners with a more challenging perceptual task than the standard method might undermine confidence–accuracy calibration by increasing examiner overconfidence in their judgments.

In two experiments, we examined confidence–accuracy calibration among mock forensic examiners who used either the filler-control method or the standard method to analyze forensic fingerprint evidence. Participants using the filler-control method compared a latent fingerprint to an evidence lineup consisting of four fingerprints—one suspect print and three filler prints—and received error feedback following match judgments on filler samples. Participants using the standard procedure compared a latent fingerprint to a single suspect fingerprint and never received error feedback. To preview, the results from Experiment 1 were consistent with the hard-easy effect in an undergraduate student sample: The filler-control method increased overconfidence and reduced confidence–accuracy calibration compared to the standard procedure. In Experiment 2, we sought to replicate these findings using a sample of forensic science students.

## 2. Experiment 1

### 2.1. Method

#### 2.1.1. Design

This experiment used a 2 (analysis procedure: standard procedure vs. filler-control procedure) × 2 (trial type: matching vs. non-matching) mixed factorial design. Analysis procedure was varied between-subjects, and trial type was varied within subjects.

The experiment was not preregistered, but the research question and hypothesis were described in the second author’s undergraduate thesis prospectus prior to collecting data. This document is available on OSF at https://osf.io/vbezx (accessed on 29 August 2025).

#### 2.1.2. Participants

We recruited undergraduate students over the age of 18 who participated for credit in a psychology, statistics, or research methods course at Arizona State University. Students were ineligible to participate if they were using a mobile device. We excluded students who did not complete the study in full (*n* = 32), were unable to view the entire training video (*n* = 9), or were unable to see the study stimuli (*n* = 1). The final sample consisted of 347 students who ranged in age from 18 to 59 years old (*M* = 23.08, *SD* = 6.89). Students self-identified their race/ethnicity as White (46.4%), Hispanic or Latinx (22.8%), Multiracial (14.4%), Asian (8.1%), Black or African American (5.5%), or other (2.9%). The sample was majority female (73.8%), with 24.8% identifying as male and 1.4% identifying as other.

#### 2.1.3. Materials

We created sixteen fingerprint sets with varying levels of difficulty using fingerprints from a database created by Growns and Kukucka (2021). The database contains latent fingerprint sets that were obtained by having individuals grasp various objects (e.g., glass, plastic); the latent fingerprints were then lifted from those objects using aluminum, black, or magneto flake powder. Each latent fingerprint set has an associated matching fingerprint that was collected by having the same individual who grasped the objects place their finger on ink and then roll it on a fingerprint card. Each set also includes five non-matching ink-rolled fingerprints that were identified by the American Fingerprint Identification System (AFIS) as close ink-rolled non-matches to the latent prints.

We selected sixteen latent fingerprints and corresponding ink-rolled suspect prints for use in the current research. Eight of the ink-rolled suspect prints matched the latent fingerprint, and eight of the ink-rolled suspect prints did not match the latent fingerprint. For the filler-control condition, we selected three additional non-matching ink-rolled prints to serve as fillers. Thus, each fingerprint set consisted of one latent fingerprint and either a single suspect print (standard condition) or an “evidence lineup” of a suspect print and three filler prints that did not match the latent print but were close non-matches (filler-control condition). The suspect and filler prints were presented in a random order in both the matching and non-matching trials.

#### 2.1.4. Measures

For each trial, we collected a match judgment and corresponding confidence rating. In the standard condition, participants could select either “match” or “no match” for each fingerprint set; in the filler-control condition, participants could select one of the comparison prints as a match or select “no match.” We collected confidence using a sliding scale ranging from 0% to 100% in 10% increments.

#### 2.1.5. Procedure

Participants were recruited to participate in this online study via the research participation system at Arizona State University. They were directed to a Qualtrics survey where they were told that the study must be completed on a computer, laptop, or tablet and not a mobile phone. Participants who attempted to continue using a mobile phone were bounced from the study and asked to return using a computer, laptop or tablet. Participants using a permitted device proceeded to the consent form, which explained that the purpose of the research was to compare fingerprint sets to determine whether any of the prints match.

After consenting to participate, participants were randomly assigned to the standard condition or filler-control condition. Participants in the standard condition read the following instructions: “In this study, you will be evaluating eight pairs of fingerprints. Each pair will include one fingerprint from the crime scene and one ‘exemplar’ fingerprint. The ‘exemplar’ fingerprint is from the suspect under investigation for the crime. Your job will be to determine whether the exemplar print matches the crime scene print. After each decision, you will be asked to indicate your confidence in your judgment.”

Participants in the filler-control condition read, “In this study, you will be evaluating eight sets of fingerprints. Each set will include one fingerprint from the crime scene and four ‘exemplar’ fingerprints. Three of the four ‘exemplar’ fingerprints are from known-innocent individuals. One ‘exemplar’ fingerprint is from the suspect under investigation for the crime. Your job will be to determine whether any of the exemplar prints matches the crime scene print, and if so, which one. After each decision, you will be asked to indicate your confidence in your judgment.” All participants were then shown a two and-a-half minute training video explaining how to perform fingerprint comparison in which they were instructed how to identify arches, loops, and whorls (College & Career Ready Labs | Paxton Patterson, 2016).

Participants then evaluated eight sets of fingerprints (four matching and four non-matching) in randomized order. In the filler-control condition, the order of the four comparison fingerprints was also randomized, and participants did not know which fingerprint was the suspect print and which were filler prints. Following each decision, participants provided their confidence rating. Due to a programming error, there was a difference in the confidence-scale instructions used in the filler-control and standard conditions. For the filler-control method, participants were instructed to indicate their confidence on a scale ranging from “0 being not confident at all and 100 being very confident.” For the standard method, participants were instructed to indicate their confidence on a scale from “0% confident to 100% confident.” Participants in both conditions used the same scale (i.e., a sliding scale ranging from 0 to 100 without verbal labels or percentages) to render their confidence judgments.

Participants in the filler-control condition who made a “match” judgment on a filler fingerprint then received the following feedback on a new screen after they rendered their judgment: “The print that you chose was not the suspect’s fingerprint. It was the fingerprint of a known-innocent individual.” After judging all eight sets of fingerprints, participants responded to demographic questions assessing their gender, age, and ethnicity/race, after which they were debriefed and awarded credit for participating.

### 2.2. Results

#### 2.2.1. Match Judgments

Suspect-match, filler-match, and non-match judgments for the two procedures and corresponding diagnosticity ratios are summarized in Table 1; individual filler-match judgments are also presented in the Supplemental Materials (Table S1). The distribution of participants’ responses reveals a few descriptive insights. First, the filler-control method reduced innocent-suspect matches (i.e., suspect-match judgments in non-matching trials; 13.2%) compared to the standard method (35.7%), but it also reduced hits (i.e., suspect-match judgments in matching trials; 38.5% vs. 67.7%). This decrease in suspect-match judgments in the filler-control method compared to the standard method was not driven by an increase in non-match judgments: The filler-control method produced fewer non-match judgments than the standard method in both matching (20.7% vs. 32.3%) and non-matching (26.7% vs. 64.3%) trials. Instead, the reduction in suspect-match judgments in the filler-control method was driven by match judgments on filler samples, which occurred frequently in both matching (40.8%) and non-matching (60.1%) trials. The diagnosticity ratios for suspect-match, filler-match, and non-match judgments suggest that the filler-control method produced more diagnostic incriminating evidence (i.e., suspect-match judgments) but less diagnostic exonerating evidence (i.e., filler-match judgments and non-match judgments) compared to the standard procedure.

We conducted inferential analyses of these patterns using generalized linear mixed effects regression analyses (glmer) using the lme4 (Bates et al., 2015) and emmeans (Lenth, 2025) packages in R. We generated two models: one for suspect-match judgment accuracy and one for non-match judgment accuracy. Note that the analysis of suspect-match judgment accuracy is mathematically redundant with an analysis of all exonerating judgments (i.e., non-match and filler-match judgments) because the proportions associated with those outcomes must sum to 1.00. Thus, in addition to examining suspect-match judgment accuracy, we examined non-match judgment accuracy. Together, these models tested how the presence of fillers affected the accuracy of suspect-match judgments and non-match judgments.

In both models, we specified procedure and trial type as fixed effects and participant as a random effect. We then added the procedure × trial type interaction to test whether suspect-match judgment accuracy or non-match judgment accuracy differed between procedures. We also conducted full ROC analyses comparing the two procedures; these analyses are presented in the Supplemental Materials (Figure S1) and are consistent with the results reported here.

##### Suspect-Match Judgment Accuracy

Demonstrating that participants were able to discriminate between matching and non-matching fingerprint sets, participants made significantly more suspect-match judgments on matching trials (52.88%) than on non-matching trials (24.28%), *B* = 1.36, *SE* = 0.09, *z* = 15.64, *p* < .001, *OR* = 3.91, 95% CI: [3.30, 4.64]. Participants also made significantly more suspect-match judgments in the standard procedure (51.68%) than in the filler-control procedure (25.85%), *B* = −1.24, *SE* = 0.09, *z* = −14.30, *p* < .001, *OR* = 0.29, 95% CI [0.24, 0.34]. The procedure × trial type interaction was not significant, *B* = 0.08, *SE* = 0.18, *z* = 0.48, *p* = 0.63, *OR* = 1.09, 95% CI [0.77, 1.54]. In other words, the two procedures did not differ in the capacity to discriminate between matching and non-matching fingerprint sets based on suspect-match judgments.

##### Non-Match Judgment Accuracy

Again demonstrating that participants were able to discriminate between matching and non-matching fingerprint sets, participants made significantly more non-match judgments on non-matching trials (45.24%) than on matching trials (26.44%), *B* = −0.92, *SE* = 0.09, *z* = −10.63, *p* < .001, *OR* = 0.40, 95% CI [0.34, 0.47]. Participants also made significantly more non-match judgments in the standard procedure (48.32%) than in the filler-control procedure (23.72%), *B* = −1.19, *SE* = 0.10, *z* = −12.28, *p* < .001, *OR* = 0.30, 95% CI [0.25, 0.37]. These effects were qualified by a significant two-way interaction between procedure and trial type, indicating that non-match judgment discriminability differed between procedures, *B* = 1.03, *SE* = 0.17, *z* = 5.97, *p* < .001, *OR* = 2.81, 95% CI [2.00, 3.95]. Specifically, non-match judgments in the standard procedure better discriminated between matching and non-matching fingerprint sets (32.31% vs. 64.33%), *B* = 1.38, *SE* = 0.12, *z* = 11.71, *p* < .001, *OR* = 3.95, 95% CI [3.14, 4.98], than did non-match judgments in the filler-control procedure (20.74% vs. 26.70%), *B* = 0.34, *SE* = 0.13, *z* = 2.67, *p* = .008, *OR* = 1.41, 95% CI [1.09, 1.81]. Thus, the standard procedure was associated with a superior capacity to discriminate between matching and non-matching fingerprint sets based on non-match judgments. This is also consistent with the pattern observed in the ROC analysis that we present in Supplemental Materials (Figure S1).

#### 2.2.2. Confidence–Accuracy Calibration

**Figure 1:**
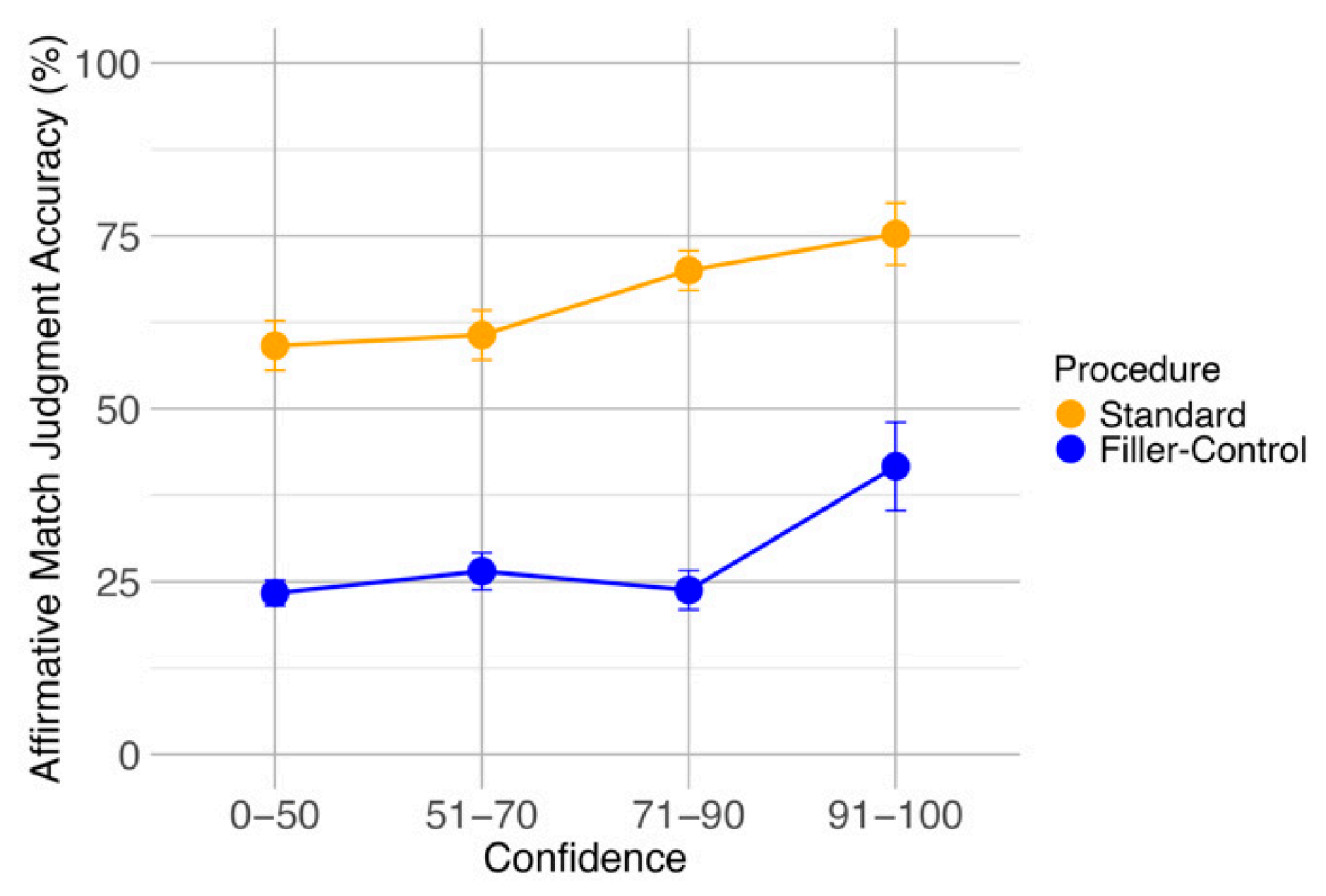
Experiment 1 Calibration Curves for Affirmative Match Judgments in the Standard and Filler-Control Procedures. Note. Error bars represent standard errors.

**Figure 2:**
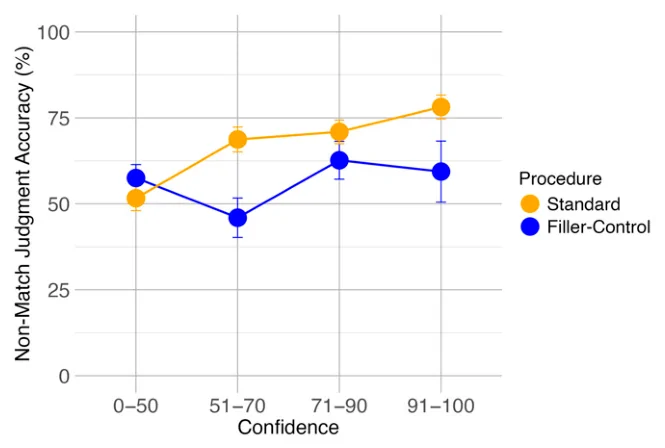
Experiment 1 Calibration Curves for Non-Match Judgments in the Standard and Filler-Control Procedures. Note. Error bars represent standard errors.

Confidence–accuracy calibration analysis compares participants’ reported level of confidence with the proportion of accurate judgments made at that confidence level. If participants’ confidence reports are perfectly calibrated, then participants who are 100% confident should be 100% accurate, participants who are 90% confident should be 90% accurate, and so forth. In our calibration analyses, accuracy for affirmative match judgments was computed by dividing accurate affirmative match judgments (i.e., suspect-match judgments in matching trials) by all affirmative match judgments (i.e., suspect-match judgments in matching trials plus all affirmative match judgments in non-matching trials; Brewer & Wells, 2006). Accuracy for non-match judgments was computed by dividing accurate non-match judgments (i.e., non-match judgments in non-matching trials) by all non-match judgments (i.e., non-match judgments in non-matching and matching trials). Figure 1 and Figure 2 display the calibration curves for affirmative match and non-match judgments, respectively, and Table 2 and Table 3 provide the corresponding confidence statistics.

We report three confidence statistics: calibration, over/underconfidence, and resolution (see Baranski & Petrusic, 1994; Lichtenstein & Fischhoff, 1977; Yaniv et al., 1991). The calibration statistic provides an overall estimate of the calibration between confidence and accuracy and varies from 0 (perfect calibration) to 1 (no calibration). The over/underconfidence statistic (O/U) indicates the extent to which confidence reports tend to over- or underestimate accuracy. It varies from −1 to +1, with negative O/U scores denoting underconfidence (i.e., lower confidence than is warranted by accuracy) and positive O/U scores denoting overconfidence (i.e., higher confidence than is warranted by accuracy). Finally, we provide a resolution statistic, Adjusted Normalized Discrimination Index (Yaniv et al., 1991), which indicates how well confidence judgments discriminate between accurate and inaccurate judgments. It varies from 0 (no discrimination between accurate and inaccurate judgments) to 1 (perfect discrimination between accurate and inaccurate judgments). We used a bootstrapping procedure with 10,000 samples to inferentially compare the confidence statistics for the standard procedure and the filler-control procedure.

We additionally tested whether confidence–accuracy calibration improved over time for either affirmative match judgments or non-match judgments. We fit glmer models using the lme4 (Bates et al., 2015) and emmeans (Lenth, 2025) packages in R. In both models, we specified procedure, mean-centered trial order, mean-centered confidence, and all of the higher-order interactions as fixed effects and participant as a random effect, with accuracy as the outcome. If the filler-control method improves calibation through the provision of error feedback, we would expect to observe a significant three-way interaction in which the association between confidence and accuracy increased over time for the filler-control method but not for the standard method.

##### Calibration of Affirmative Match Judgments

We predicted that the filler-control procedure would improve calibration and reduce overconfidence. Contrary to our hypothesis, participants using the filler-control procedure exhibited worse confidence–accuracy calibration (0.12) than did participants using the standard procedure (0.02, *p* < .001), and overconfidence was greater in the filler-control condition (0.28) than in the standard condition (0.006, *p* < .001). Resolution did not significantly differ across the two procedures (*p* = .59). Moreover, the analysis examining confidence–accuracy calibration across trials showed no difference in the extent to which calibration changed over time in the filler-control method and the standard method, as indicated by a non-significant three-way (procedure × trial order × confidence) interaction on affirmative match judgment accuracy, *B* = −0.001, *SE* = 0.002, *z* = −0.49, *p* = .63, *OR* = 0.999, 95% CI [0.995, 1.003].

##### Calibration of Non-Match Judgments

Calibration analyses for non-match judgments again indicated that participants using the filler-control procedure exhibited worse confidence–accuracy calibration (0.05) than did participants using the standard procedure (0.02, *p* = .007). Neither over/underconfidence (*p* = .83) nor resolution (*p* = .18) significantly differed between procedures for non-match judgments. Moreover, the analysis examining confidence–accuracy calibration across trials showed no difference in the extent to which calibration changed over time in the filler-control method and the standard method, as indicated by a non-significant three-way (procedure × trial order × confidence) interaction on non-match judgment accuracy, *B* = 0.002, *SE* = 0.003, *z* = 0.99, *p* = .32, *OR* = 1.002, 95% CI [0.998, 1.007].

#### 2.2.3. Confidence–Accuracy Characteristic Analysis

The above calibration analyses provide a measure of psychological calibration, or the ability of participants to scale their confidence with their accuracy for all judgments—namely, affirmative match judgments on suspect and filler samples and non-match judgments. However, because match judgments on the suspect’s sample are highly legally consequential, there is value in examining the extent to which confidence is associated with accuracy for suspect-match judgments specifically. Confidence–Accuracy Characteristic (CAC) analysis was designed specifically to do that, albeit in the eyewitness-identification context (Mickes, 2015). CAC analysis plots the accuracy of affirmative responses to the suspect (or suspect’s sample) across confidence levels, thus providing a measure of a procedure’s positive predictive value (PPV) across confidence levels. Positive predictive value indicates how likely it is that someone with a positive test result (e.g., an eyewitness’s identification of the suspect or a forensic examiner’s match judgment on the suspect’s sample) truly has the outcome of interest (i.e., the suspect is the culprit; Smith et al., 2017). In the current context, PPV refers to the probability that a suspect-match judgment is accurate, which we will refer to as suspect-match judgment accuracy.

We generated CAC plots for the filler-control procedure by dividing the number of accurate suspect-match judgments (i.e., suspect-match judgments in matching trials) by all suspect-match judgments (i.e., suspect-match judgments in matching and non-matching trials) at a given confidence level. Note that CAC plots for the standard procedure are the same as confidence–accuracy plots because the only match judgments possible are suspect-match judgments. Readers should note that the values shown here assume a 50% base rate of match-present trials. We were interested in conducting a relative comparison of PPV between the two procedures rather than estimating absolute levels of suspect-match judgment accuracy, which are dependent on the base rate of match presence. The Supplemental Materials present PPV plots at varying base rates of match presence (25%, 50%, and 75%), but the relative order of the curves does not change (Figure S2).

**Figure 3:**
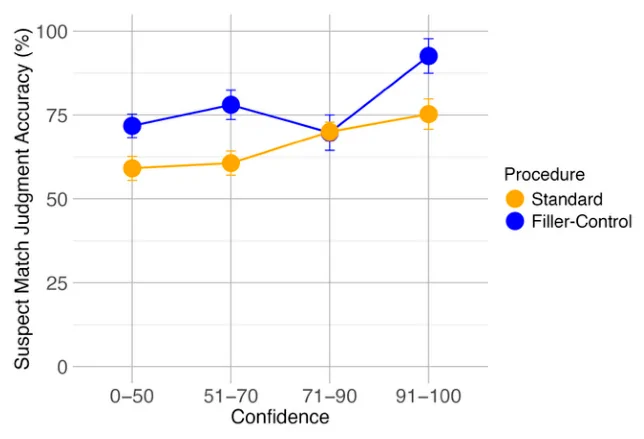
Experiment 1 Confidence–Accuracy Characteristic Curves Across Procedures. Note. Error bars represent standard errors.

As shown in Figure 3, the filler-control procedure tended to be associated with greater PPV across confidence levels. Using a bootstrapping procedure with 10,000 samples, inferential analyses of PPV for each of the four confidence bins (see Table 4) indicated that this difference was significant for the 0–50 confidence bin (*p* = .013), the 51–70 confidence bin (*p* = .003), and the 91–100 confidence bin (*p* = .017), but not for the 71–90 confidence bin (*p* = .99). It is noteworthy that the filler-control procedure was associated with higher PPV at the 91–100% confidence level (92.6% suspect-match judgment accuracy) compared to the standard procedure (75.3% suspect-match judgment accuracy); this indicates that the filler-control method provided more accurate suspect-match judgments at the highest levels of mock examiner confidence.

### 2.3. Discussion

In Experiment 1, we tested whether the filler-control method reduces examiner overconfidence and improves confidence–accuracy calibration relative to the standard forensic analysis procedure. Contrary to our predictions, the filler-control method resulted in greater overconfidence and worse confidence–accuracy calibration relative to the standard procedure. Moreover, non-match judgment accuracy was significantly higher in the standard procedure than in the filler-control procedure. That is because the presence of fillers substantially reduced the percentage of accurate non-match judgments (i.e., non-match judgments in non-matching trials)—from 64.3% to 26.7% (a 37.6% reduction)—without a correspondingly large reduction in inaccurate non-match judgments (i.e., non-match judgments in matching trials; 32.3% vs. 20.7%; an 11.6% reduction). Even though filler-match judgments were more frequent in non-matching trials than in matching trials—indicating that filler-match judgments are diagnostic of innocence—neither filler-match judgments (DR = 1.47) nor non-match judgments (DR = 1.29) in the filler-control procedure were as diagnostic of innocence as were non-match judgments in the standard procedure (DR = 1.99).

Although the filler-control procedure was associated with worse confidence–accuracy calibration and decreased non-match judgment accuracy compared to the standard procedure, it resulted in superior PPV relative to the standard procedure. In other words, suspect-match judgments were more accurate in the filler-control procedure than in the standard procedure across most confidence levels, including for suspect-match judgments made with the highest level of confidence. These findings indicate that, for consequential incriminating outcomes (match judgments on the suspect’s sample) the filler-control method produced more reliable evidence than did the standard procedure.

Because forensic fingerprint analysis in the field requires training, it is possible that our sample of undergraduate students is not representative of practicing forensic fingerprint examiners. To increase the generalizability of our findings, we aimed to replicate the results of Experiment 1 using a more representative sample—namely, a sample of forensic science students. Experiment 2 was thus identical to Experiment 1, except that we recruited forensic science students with relevant training and/or experience in forensic analysis.

## 3. Experiment 2

### 3.1. Method

#### 3.1.1. Design

This experiment used a 2 (analysis procedure: standard procedure vs. filler-control procedure) × 2 (trial type: matching vs. non-matching) mixed factorial design. Analysis procedure was varied between-subjects, and trial type was varied within subjects. The study methods were identical to those used in Experiment 1, except that we recruited a sample of forensic science students and collected demographic information pertaining to the students’ training and experience in forensic fingerprint analysis.

#### 3.1.2. Participants

We recruited undergraduate and graduate forensic science students over the age of 18 at Arizona State University. In exchange for participation, students were entered into a drawing to win one of 20 $50 Amazon gift cards. Students were ineligible to participate if they were using a mobile device. We excluded students who did not complete the study in full (*n* = 37) or were unable to view the entire training video (*n* = 7). The final sample consisted of 105 students who ranged in age from 18 to 51 years old (*M* = 23.40, *SD* = 6.78). Students self-identified their race/ethnicity as White (54.8%), Hispanic or Latinx (23.1%), Multiracial (11.5%), Asian (5.8%), Black or African American (2.9%), American Indian or Alaska Native (1.0%), or other (1.0%). The sample was majority female (86.5%), with 13.5% identifying as male.

Most of the participants (56.2%) were enrolled in an in-person Forensic Science Bachelor’s program, with 25.7% enrolled in an online Forensic Science Bachelor’s program, 14.3% enrolled in an online Professional Masters in Forensic Science program, and 3.8% enrolled in an online Masters in Forensic Science program. At the time of participation, participants had completed nearly three semesters in their academic program (*M* = 2.90, *SD* = 2.12). Most participants (98.1%) had never been employed as a forensic fingerprint examiner, though two (1.9%) had. Participants reported having received varying levels of training in forensic fingerprint analysis prior to completing the experiment, with 44.8% reporting having received no training, 33.3% reporting having received a little training, 18.1% reporting having received some training, and 3.8% reporting having received a lot of training.

### 3.2. Results

#### 3.2.1. Match Judgments

Suspect-match, filler-match, and non-match judgments for the two procedures and corresponding diagnosticity ratios are summarized in Table 5; individual filler-match judgments are also presented in the Supplemental Materials (Table S2). We again begin with a descriptive comparison of the distribution of participants’ responses. The patterns parallel those observed in Experiment 1. The filler-control method reduced innocent-suspect-matches (i.e., suspect-match judgments in non-matching trials; 9.7%) compared to the standard method (29.9%), but it also reduced hits (i.e., suspect-match judgments in matching trials; 44.9% vs. 66.5%). This decrease in suspect-match judgments in the filler-control method compared to the standard method was not driven by an increase in non-match judgments: The filler-control method produced fewer non-match judgments than the standard method in both matching (19.9% vs. 33.5%) and non-matching (37.2% vs. 70.1%) trials. Instead, the reduction in suspect-match judgments in the filler-control method was driven by filler-match judgments, which occurred frequently in both matching (35.2%) and non-matching (53.1%) trials. The diagnosticity ratios for suspect-match, filler-match, and non-match judgments again suggest that the filler-control method produced more diagnostic incriminating evidence (i.e., suspect-match judgments) but less diagnostic exonerating evidence (i.e., filler-match judgments and non-match judgments) compared to the standard procedure.

We conducted inferential analyses of these patterns using generalized linear mixed effects regression analyses (glmer) of suspect-match judgment accuracy and non-match judgment accuracy using the lme4 (Bates et al., 2015) and *emmeans* (Lenth, 2025) packages in R. As in Experiment 1, we specified procedure and trial type as fixed effects and participant as a random effect, with suspect-match judgments or non-match judgments as the outcome. We then added the procedure × trial type interaction to each model to test whether suspect-match judgment accuracy or non-match judgment accuracy differed between procedures. We also conducted full ROC analyses comparing the two procedures; these analyses are presented in the Supplemental Materials (Figure S3) and are consistent with the results of the regression analyses.

##### Suspect-Match Judgment Accuracy

The findings for suspect-match judgments replicated the findings from Experiment 1. Participants were again able to discriminate between matching and non-matching fingerprint sets, as evidenced by participants making significantly more suspect-match judgments on matching trials (56.43%) than on non-matching trials (20.48%), *B* = 1.83, *SE* = 0.18, *z* = 10.47, *p* < .001, *OR* = 6.26, 95% CI [4.44, 8.82]. Participants also made significantly more suspect-match judgments in the standard procedure (48.21%) than in the filler-control procedure (27.30%), *B* = −1.14, *SE* = 0.20, *z* = −5.58, *p* < .001, *OR* = 0.32, 95% CI [0.21, 0.48]. The procedure × trial type interaction was not significant, *B* = 0.50, *SE* = 0.36, *z* = 1.40, *p* = .163, *OR* = 1.64, 95% CI [0.82, 3.31]. In other words, the two procedures again did not differ in the capacity to discriminate between matching and non-matching fingerprint sets based on suspect-match judgments.

##### Non-Match Judgment Accuracy

Consistent with the findings from Experiment 1, participants were also able to discriminate between matching and non-matching fingerprint sets, as evidenced by significantly more non-match judgments on non-matching trials (54.76%) than on matching trials (27.14%), *B* = −1.37, *SE* = 0.16, *z* = −8.34, *p* < .001, *OR* = 0.26, 95% CI [0.19, 0.35]. Participants also made significantly more non-match judgments in the standard procedure (51.79%) than in the filler-control procedure (28.57%), *B* = −1.18, *SE* = 0.21, *z* = −5.74, *p* < .001, *OR* = 0.31, 95% CI [0.21, 0.46]. These effects were again qualified by a significant two-way interaction between procedure and trial type, indicating that non-match judgment discriminability differed between procedures, *B* = 0.72, *SE* = 0.32, *z* = 2.25, *p* = .02, *OR* = 2.06, 95% CI [1.10, 3.87]. As observed in Experiment 1, non-match judgments in the standard procedure better discriminated between matching and non-matching fingerprint sets (33.48% vs. 70.09%), *B* = 1.67, *SE* = 0.22, *z* = 7.72, *p* < .001, *OR* = 5.31, 95% CI [3.48, 8.12], than did non-match judgments in the filler-control procedure (37.24% vs. 19.90%), *B* = 0.95, *SE* = 0.24, *z* = 3.91, *p* < .001, *OR* = 2.58, 95% CI [1.60, 4.14]. Thus, the standard procedure was associated with a superior capacity to discriminate between matching and non-matching fingerprint sets based on non-match judgments.

#### 3.2.2. Confidence–Accuracy Calibration

**Figure 4:**
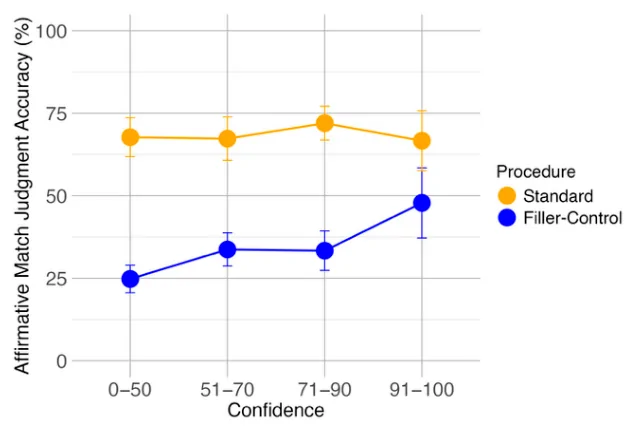
Experiment 2 Calibration Curves for Affirmative Match Judgments in the Standard and Filler-Control Procedures. Note. Error bars represent standard errors.

**Figure 5:**
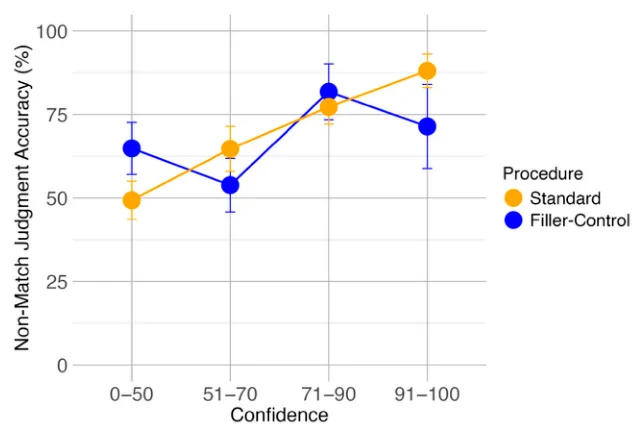
Experiment 2 Calibration Curves for Non-Match Judgments in the Standard and Filler-Control Procedures. Note. Error bars represent standard errors.

Figure 4 and Figure 5 display the calibration curves for match and non-match judgments, respectively, and Table 6 and Table 7 provide the corresponding confidence statistics.

##### Calibration of Affirmative Match Judgments

Replicating the findings from Experiment 1, participants using the filler-control procedure exhibited worse confidence–accuracy calibration (0.10) than did participants using the standard procedure (0.05, *p* = .042), and overconfidence was greater in the filler-control condition (0.28) than in the standard condition (−0.05, *p* < .001). Resolution did not significantly differ across the two procedures (*p* = .52). Again, the analysis examining confidence–accuracy calibration across trials showed no difference in the extent to which calibration changed over time in the filler-control method and the standard method, as indicated by a non-significant three-way (procedure × trial order × confidence) interaction on affirmative match judgment accuracy, *B* = 0.004, *SE* = 0.004, *z* = 0.95, *p* = .34, *OR* = 1.004, 95% CI [0.996, 1.012].

##### Calibration of Non-Match Judgments

Calibration analyses for non-match judgments again indicated that participants using the filler-control procedure exhibited worse confidence–accuracy calibration (0.05) than did participants using the standard procedure (0.01, *p* = .037). Neither over/underconfidence (*p* = .63) nor resolution (*p* = .34) significantly differed between procedures for non-match judgments. Again, the analysis examining confidence–accuracy calibration across trials showed no difference in the extent to which calibration changed over time in the filler-control method and the standard method, as indicated by a non-significant three-way (procedure × trial order × confidence) interaction on non-match judgment accuracy, *B* = 0.003, *SE* = 0.005, *z* = 0.60, *p* = .55, *OR* = 1.003, 95% CI [.994, 1.012].

#### 3.2.3. Confidence–Accuracy Characteristic Analysis

**Figure 6:**
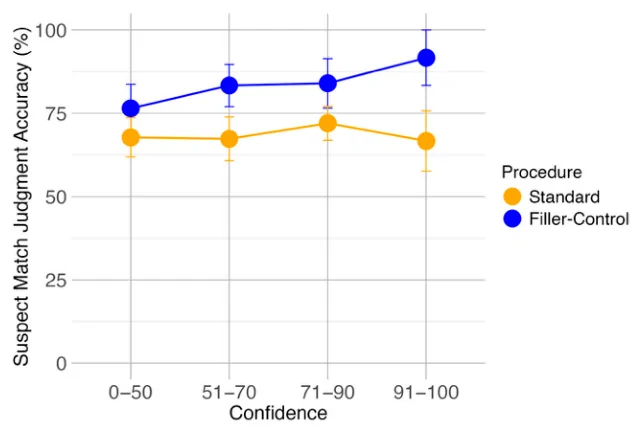
Experiment 2 Confidence–Accuracy Characteristic Curves in the Standard and Filler-Control Procedures. Note. Error bars represent standard errors.

As shown in Figure 6, the filler-control procedure again tended to be associated with greater PPV across confidence levels. Using a bootstrapping procedure with 10,000 samples, inferential analyses of PPV (see Table 8) indicated that this difference was nonsignificant across each of the four confidence bins (all *p*’s ≥ .059). Critically, however, the largest difference in PPV was observed for the highest confidence bin (91–100% confidence), where suspect-match judgment accuracy was substantially higher in the filler-control procedure (91.7%) than in the standard procedure (66.7%). Thus, consistent with Experiment 1, the filler-control method tended to produce higher suspect-match judgment accuracy than the standard procedure, especially at the highest confidence levels. As in Experiment 1, the relative order of the curves did not change across different levels of the base rate of match presence (see Figure S4 of Supplemental Materials).

### 3.3. Discussion

Experiment 2 largely replicated the findings from Experiment 1, this time in a sample of forensic science students, most of whom had previously received some training in forensic fingerprint analysis. As in Experiment 1, the filler-control method was associated with greater overconfidence and worse confidence–accuracy calibration compared to the standard procedure, and non-match judgments were more accurate in the standard procedure than in the filler-control procedure. Nevertheless, CAC analyses again indicated that the filler-control procedure was associated with superior PPV relative to the standard procedure, though the inferential comparisons across confidence bins did not achieve the conventional significance level of .05. These non-significant trends may have been attributable to low power: The forensic science student sample collected in Experiment 2 was just a third of the size of the undergraduate student sample collected in Experiment 1. Indeed, differences in PPV between the two procedures were similar across the two experiments, with some evidence of even larger effects at the highest levels of confidence among forensic science students than among our general undergraduate student sample.

## 4. General Discussion

We tested the claim that the filler-control method reduces examiner overconfidence compared to the standard forensic analysis method (Wells et al., 2013). Across two experiments involving undergraduate students (Experiment 1) and forensic science students (Experiment 2), we found evidence of the opposite effect: The filler-control method resulted in greater overconfidence and worse confidence–accuracy calibration compared to the standard method. Why did the filler-control method fail to curb mock examiners’ overconfidence? Theoretically, the capacity of the filler-control method to provide immediate error feedback should enable examiners to calibrate subjective confidence with objective accuracy over time (e.g., Arkes et al., 1987; Haddara & Rahnev, 2022). Indeed, our experiments provided ample opportunity for mock examiners to learn from their mistakes: Mock examiners using the filler-control method made filler-match judgments in approximately half of the trials and thus received error feedback frequently. Yet, even in analyses examining confidence–accuracy calibration across trials, there was no evidence of improvement in calibration in the filler-control method compared to the standard method.

The filler-control method’s potential to curb overconfidence via the provision of error feedback might be counteracted by another feature of the method: It presents an objectively more difficult task than the standard method because the presence of fillers adds “noise,” thereby complicating signal detection (Macmillan, 2002). Indeed, overall accuracy rates (hits and correct rejections) were lower in the filler-control method than in the standard method in both experiments. Our findings are therefore consistent with the hard-easy effect, the phenomenon in which overconfidence increases as task difficulty increases (Lichtenstein & Fischhoff, 1977; Lichtenstein et al., 1981). It may be the case that the increased difficulty of the filler-control method compared to the standard method overpowered any benefits afforded by the provision of immediate error feedback to mock-examiners.

There are additional features that differentiate the filler-control method and the standard forensic analysis method and could drive differences in performance. For example, the two methods might promote different decision strategies among examiners. Preliminary research suggests that the filler-control method makes examiners more conservative in their judgments, presumably to avoid making a known false positive error (e.g, Kukucka et al., 2020). Kukucka and colleagues observed this effect only for inconclusive judgments, however; non-match judgments were less frequent in the filler-control method than in the standard method, as was the case in our data. As we discuss in the Limitations section, future research that examines confidence–accuracy calibration when examiners have the option to render inconclusive judgments will be valuable.

The filler-control method might also promote the use of relative judgments, in which examiners compare the evidence samples to one another to determine the best match. Longstanding theories of eyewitness identification posit that the simultaneous presentation of lineup members promotes the use of relative judgments compared to when lineup members are presented individually (e.g., Wells, 1984, 1993). However, recent research suggests that eyewitness decision-making from lineups may be better characterized by absolute-judgment models, in which eyewitnesses compare each lineup member individually to their memory (Smith et al., 2024). Moreover, relative judgments may be limited in forensic decision-making because of the nature of forensic feature comparison. Unlike in eyewitness lineups, where eyewitnesses can often rely on rapid, holistic comparisons of lineup members’ faces, forensic examiners may need to more deliberatively compare features of each comparison sample to the crime scene sample, consistent with an absolute-judgment strategy. Nevertheless, future research could investigate the decision strategies examiners use when analyzing forensic evidence using the filler-control method versus the standard method.

### 4.1. The Incriminating-Exonerating Tradeoff of the Filler-Control Method

Across both of our experiments, the filler-control method was associated with inferior exonerating value but superior incriminating value compared to the standard method. This incriminating-exonerating tradeoff was evident in the diagnosticity ratios associated with mock-examiners’ judgments, regression analyses of non-match judgment accuracy, and PPV analyses and was confirmed by ROC analyses presented in the Supplemental Materials.

Why was the filler-control method associated with superior incriminating value but inferior exculpatory value compared to the standard method? A primary benefit of the filler-control method appears to be its potential to protect innocent suspects. Surrounding the suspect’s sample with known-innocent filler samples resulted in a larger proportional reduction in innocent-suspect-match judgments than in guilty-suspect-match judgments. This reduction in suspect-match judgments was not driven by an increase in non-match judgments; it was driven by match judgments on fillers, consistent with a differential filler siphoning mechanism. Originally demonstrated in the context of eyewitness identification from police lineups, differential filler siphoning improves the diagnostic value of incriminating outcomes and enhances PPV (e.g., Smith et al., 2017; Wells et al., 2015).

However, this benefit of the filler-control method came at a cost. Aside from impairing confidence–accuracy calibration, the filler-control method reduced non-match judgment accuracy compared to the standard method: fillers reduced accurate non-match judgments more than they reduced inaccurate non-match judgments, decreasing the diagnostic value of non-match judgments and impairing NPV. Although filler-match judgments themselves are diagnostic of innocence (i.e., they are more likely to occur in non-matching than matching trials), they were less diagnostic than were non-match judgments in the standard method. We speculate that the decreased diagnosticity of exonerating outcomes in the filler-control method (filler-match judgments and non-match judgments) compared to exonerating judgments in the standard method (non-match judgments) reflects the effects of task difficulty. By increasing the amount of noise present in the signal-detection task (e.g., Macmillan, 2002), fillers undermined decision-making accuracy overall (i.e., lower hits and correct rejections). This accuracy reduction was offset for incriminating outcomes—through a substantial decrease in innocent-suspect matches—but was not offset for exonerating outcomes.

There is yet another aspect of the filler-control method that might reduce its exculpatory value compared to the standard method. Specifically, the evidentiary value of examiners’ confidence ratings in exonerating outcomes may be lower in the filler-control method than in the standard method. In the standard method, an examiner’s confidence in an exculpatory outcome (i.e., a non-match judgment) always provides direct information about the degree to which the suspect’s sample matches the crime scene sample. In the filler-control method, however, an examiner’s confidence in an exculpatory outcome (i.e., a match decision on a filler sample or a non-match judgment) provides only indirect information about the degree to which the suspect’s sample matches the crime scene sample (e.g., Smith & Ayala, 2021). If the examiner renders a match judgment on a filler, their confidence scales the extent to which the filler sample matches the crime scene sample. And if the examiner makes a non-match judgment, the meaning of confidence is ambiguous: It might scale the average extent to which all of the comparison samples match the crime scene sample or the extent to which the closest-to-matching comparison sample matches the crime scene sample (e.g., Lindsay et al., 2013; Weber & Brewer, 2006). Either way, confidence in a non-match judgment likewise does not directly scale the match between the suspect sample and the crime scene sample. As a result, the additional information gleaned from examiners’ confidence is less directly informative about the likely guilt of the suspect in the filler-control method than in the standard method.

### 4.2. Alternative Approaches to Forensic-Feature Comparison

Our findings suggest that neither the filler-control method nor the standard forensic analysis method is an objectively superior method of analyzing forensic evidence. This observation underscores the need for a new approach that better maximizes both the incriminating and exonerating value of forensic evidence. The filler-control method offers multiple advantages that are worth preserving—namely, the ability to neutralize contextual bias, expose invalid techniques and fraudulent analysts, produce error rate estimates for a given technique, laboratory, and analyst, and provide error feedback to analysts (Wells et al., 2013). Our data also demonstrate that the filler-control method can substantially reduce innocent-suspect-match judgments, suggesting that the method may be especially valuable in settings that run a high risk of false positive errors. To enhance its exonerating potential, the filler-control method could be refined to capture more nuanced information following exculpatory judgments. Borrowing again from innovations in the eyewitness-identification literature, several approaches could accomplish this. These include (1) a ranking procedure in which the lineup members are ranked in terms of their match to the crime scene sample (e.g., Carlson et al., 2019; Tuttle et al., 2025); (2) a perceptual-scaling approach that measures the similarity between the crime scene sample and each comparison sample (e.g., Gepshtein et al., 2020); (3) a rule out procedure in which confidence that each comparison sample does *not* match the crime scene sample is obtained following a filler-match or non-match judgment (e.g., Ayala et al., 2022; Smith et al., 2023); and (4) a confidence-ratings procedure in which the examiner provides a confidence rating that each sample matches or does not match the crime scene sample (Sauer et al., 2008, 2012). Future research could test these procedures against the filler-control method and the standard forensic analysis method to determine which might prove superior in forensic contexts.

It remains possible, however, that the costs associated with the filler-control method will ultimately outweigh its benefits. An alternative approach with considerable promise has been proposed by Guyll and Madon (2023). In their method, a liaison serves as an intermediary between investigators and forensic examiners, preparing the evidence for analysis. Specifically, the liaison obtains the crime scene sample and the suspect’s sample from investigators and provides them to the examiner along with an additional, case-matched mock evidence pair. In a case involving fingerprint evidence, for example, the liaison would assemble the latent print and suspect print pair from the case as well as a second pair consisting of a mock-latent print and a mock-suspect print that may or may not match one another. The forensic examiner would not know which pair is from the case nor the ground truth of the mock pair (i.e., whether they match). Hence, the examiner would produce two judgments: one for the case and a separate, independently verifiable judgment.

This approach has the potential to preserve the benefits of the filler-control method while addressing its limitations. First, it could reduce innocent-suspect matches, as examiners would know that a false positive on the mock pair would be detected. Second, it protects against contextual bias because even if examiners are exposed to contextual case information (which the liaison should often prevent), they would not know which pair the information applies to. Third, the method would expose invalid techniques and fraudulent analysts, who would make detectable errors on the mock evidence. Fourth, over time, this process would generate error rate estimates for the examiner, lab, and technique. Fifth, the approach allows for feedback on examiner performance, potentially facilitating an improvement in confidence–accuracy calibration over time. Moreover, unlike the filler-control method—where fillers increase task difficulty and undermine performance—the mock and case judgments are made independently, preserving performance advantages of the standard method. While this procedure has yet to undergo empirical testing, we consider it a promising and innovative path for future forensic practice.

### 4.3. Limitations

As an initial investigation of confidence–accuracy calibration in the forensic filler-control method, the current research had several limitations, the most notable of which concerns the generalizability of the samples. In Experiment 1, participants were undergraduate students with no prior training in forensic evidence analysis—a population that differs in meaningful ways from forensic professionals. Although we attempted to address this limitation by showing all participants a training video before they began the task and by recruiting forensic science students in Experiment 2, it would be ideal to replicate the current findings with a sample of experienced forensic examiners. It is possible, for example, that professional forensic examiners are less subject to the hard-easy effect than our novice student samples. If that were the case, the filler-control method could conceivably increase forensic professionals’ confidence–accuracy calibration.

A second set of limitations relates to characteristics of the measures used in our experiments. As mentioned in Section 2.1.5, a programming error resulted in a difference in the confidence-scale instructions used in the filler-control and standard procedure conditions. For the filler-control method, participants were instructed to indicate their confidence on a scale ranging from “0 being not confident at all and 100 being very confident.” For the standard method, participants were instructed to indicate their confidence on a scale from “0% confident to 100% confident.” Critically, however, participants in both conditions used the same scale (i.e., a sliding scale ranging from 0 to 100 without verbal labels or percentages) to render their confidence judgments. Thus, although these slight differences in the wording of the instructions are not ideal, we believe that this aspect of our method likely had a trivial effect on our findings.

Another limitation of our measures is that we did not permit participants to render inconclusive judgments, which is a response option available to practicing forensic examiners (Wells et al., 2013). We imposed this constraint to preserve statistical power for our confidence–accuracy calibration analyses, which exclude inconclusive judgments. Moreover, some have argued that the inconclusive response option should be abandoned in favor of an approach that collects information about the examiner’s decision criterion (e.g., the examiner’s confidence), as the filler-control method does (Albright, 2022). Nonetheless, the widespread use of the inconclusive option in current forensic practice means that omitting it in our experiments reduces the ecological validity of our findings. For example, it is possible that the filler-control method’s ability to immediately expose errors encourages a more conservative response style, leading professionals to render inconclusive judgments more often (e.g., Kukucka et al., 2020; see also Miller, 1987). Shifting examiners’ responses in this way could conceivably influence their confidence–accuracy calibration. To the extent that examiners render inconclusive judgments on judgment tasks they perceive as difficult (e.g., Albright, 2022), it could moderate the hard-easy effect, thereby reducing overconfidence. Thus, it will be important for future research to replicate these findings using a paradigm that includes an inconclusive option.

## 5. Conclusions

We conducted the first investigation of the proposition that the filler-control method improves confidence calibration relative to the standard forensic analysis method. Not only did the filler-control method fail to improve confidence–accuracy calibration, it produced less reliable exculpatory evidence than the standard method. On the other hand, the filler-control method produced more reliable incriminating evidence, which is especially valuable given the role of false suspect-match judgment evidence in convictions of the innocent (Morgan, 2023). Our findings, therefore, suggest opportunities for developing alternative forensic analysis methods to maximize the potential of forensic evidence to incriminate guilty suspects while protecting innocent suspects from the risk of wrongful conviction.

## Figures and Tables

**Table 1:** Experiment 1 Match Judgments Across Procedures.

	Filler-Control Procedure			Standard Procedure	
	Suspect-Match Judgment	Filler-Match Judgment	Non-Match Judgment	Suspect-Match Judgment	Non-Match Judgment
Matching Trial	38.49%	40.77%	20.74%	67.69%	32.31%
Non-Matching Trial	13.21%	60.09%	26.70%	35.67%	64.33%
DR	2.91	1.47	1.29	1.90	1.99

Note. DR = Diagnosticity Ratio. DRs for suspect-match judgments were calculated by dividing the suspect-match judgment rate in matching trials by the suspect-match judgment rate in non-matching trials. DRs for filler-match judgments and non-match judgments were calculated by dividing the filler-match judgment and non-match judgment rates in non-matching trials by the filler-match judgment and non-match judgment rates in matching trials, respectively.

**Table 2:** Experiment 1 confidence statistics for affirmative match judgments across procedures.

Procedure	Calibration	Over/Underconfidence	Resolution
Standard	0.02 *	0.006 *	0.01
Filler-Control	0.12 *	0.28 *	0.007

Note. * *p* < 0.001; indicates significant difference between procedures.

**Table 3:** Experiment 1 confidence statistics for non-match judgments across procedures.

Procedure	Calibration	Over/Underconfidence	Resolution
Standard	0.02 *	0.0007	0.04
Filler-Control	0.05 *	−0.007	0.005

Note. * *p* < 0.01; indicates significant difference between procedures.

**Table 4:** Experiment 1 Confidence–Accuracy Characteristic Statistics.

		95% CI		
	Effect Size	Lower	Upper	*p*
0–50 Bin	0.126	0.027	0.225	0.013
51–70 Bin	0.174	0.063	0.283	0.003
71–90 Bin	−0.001	−0.121	0.113	0.990
91–100 Bin	0.172	0.031	0.298	0.017

**Table 5:** Experiment 2 Match Judgments Across Procedures.

	Filler-Control Procedure			Standard Procedure	
	Suspect-Match Judgment	Filler-Match Judgment	Non-Match Judgment	Suspect-Match Judgment	Non-Match Judgment
Matching Trial	44.90%	35.20%	19.90%	66.52%	33.48%
Non-Matching Trial	9.69%	53.06%	37.24%	29.91%	70.09%
DR	4.63	1.51	1.87	2.22	2.09

Note. DR = Diagnosticity Ratio. DRs for suspect-match judgments were calculated by dividing the suspect-match judgment rate in matching trials by the suspect-match judgment rate in non-matching trials. DRs for filler-match judgments and non-match judgments were calculated by dividing filler-match judgment and non-match judgment rates in non-matching trials by filler-match judgment and non-match judgment rates in matching trials, respectively.

**Table 6:** Experiment 2 confidence statistics for affirmative match judgments across procedures.

Procedure	Calibration	Over/Underconfidence	Resolution
Standard	0.05 *	−0.05 **	−0.01
Filler-Control	0.10 *	0.28 **	0.009

Note. * *p* < 0.05, ** *p* < 0.001; indicates significant difference between procedures.

**Table 7:** Experiment 2 confidence statistics for non-match judgments across procedures.

Procedure	Calibration	Over/Underconfidence	Resolution
Standard	0.01 *	−0.03	0.08
Filler-Control	0.05 *	−0.06	0.02

Note. * *p* < 0.05; indicates significant difference between procedures.

**Table 8:** Experiment 2 Confidence–Accuracy Characteristic Statistics.

		95% CI		
	Effect Size	Lower	Upper	*p*
0–50 Bin	0.087	−0.101	0.272	0.371
51–70 Bin	0.161	−0.022	0.338	0.079
71–90 Bin	0.120	−0.063	0.288	0.195
91–100 Bin	0.250	−0.007	0.481	0.059

## Data Availability

The original data presented in both studies are openly available on OSF at https://osf.io/zxhjn/files/osfstorage, accessed on 29 August 2025. Please note that information about participants’ academic programs has been removed from the Experiment 2 dataset to prevent participant identification.

## Supplementary Materials

The following supporting information can be downloaded at: https://www.mdpi.com/article/10.3390/bs15091191/s1, Table S1: Experiment 1 Breakdown of Individual Filler-Match, Suspect-Match, and Non-Match Rates; Table S2: Experiment 2 Breakdown of Individual Filler-Match, Suspect-Match, and Non-Match Rates; Figure S1: Experiment 1 Investigator Full ROCs for the Standard and Filler-Control Procedures; Figure S2: Experiment 1 Confidence-Accuracy Characteristic Curves in the Standard and Filler-Control Procedures at Varying Base Rates of Match Presence; Figure S3: Experiment 2 Investigator Full ROCs for the Standard and Filler-Control Procedures; Figure S4: Experiment 2 Confidence-Accuracy Characteristic Curves in the Standard and Filler-Control Procedures at Varying Base Rates of Match Presence. (Robin et al., 2011; Smith & Neal, 2021; Yang & Smith, 2023; Smith et al., 2020b) are cited in the supplementary materials.

## Abbreviations

The following abbreviations are used in this manuscript:

O/U	Over/underconfidence statistic
PPV	Positive predictive value
NPV	Negative predictive value
