# Correction: Rath et al. (2025). Does the Forensic Filler-Control Method Reduce Examiner Overconfidence? An Experimental Investigation Using Mock Fingerprint Examiners. *Behavioral Sciences*, *15*(9), 1191

**DOI:** 10.3390/bs16091638

**Published:** 2026-09-14

**Authors:** Hannah J. Rath, Bethany Rocha, Andrew M. Smith, Laura Smalarz

**Affiliations:** 1 School of Interdisciplinary Forensics, Arizona State University, Glendale, AZ 85306, USAlaura.smalarz@asu.edu (L.S.); 2 Department of Psychology, Iowa State University, Ames, IA 50011, USA

## Error in Figures

In the original publication (Rath et al., 2025), there were some mistakes in Figures 1–6, S2 and S4 as published. Incorrect error bars appeared in the figures. The corrected “Figure 1, Figure 2, Figure 3, Figure 4, Figure 5, Figure 6, Figure S2 and Figure S4” appear below. 

## Error in Tables

In the original publication (Rath et al., 2025), there were some mistakes in Tables 2, 3, 6 and 7 as published. The values for adjusted resolution were not adjusted properly. The corrected “Table 2, Table 3, Table 6 and Table 7” appear below. 

The authors state that the scientific conclusions are unaffected. This correction was approved by the Academic Editor. The original publication has also been updated.

## Figures and Tables

**Figure 1:**
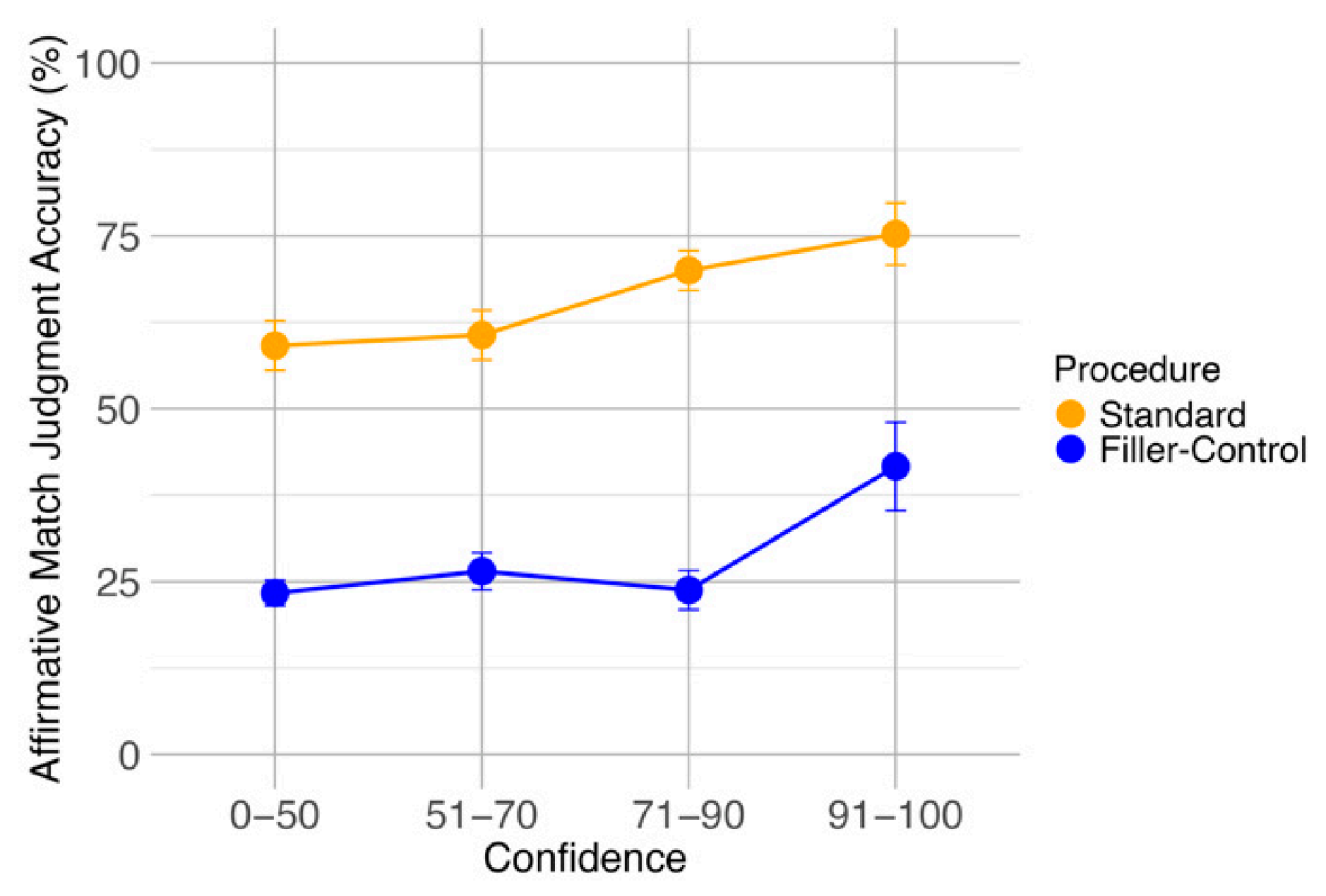
Experiment 1 Calibration Curves for Affirmative Match Judgments in the Standard and Filler-Control Procedures. Note. Error bars represent standard errors.

**Figure 2:**
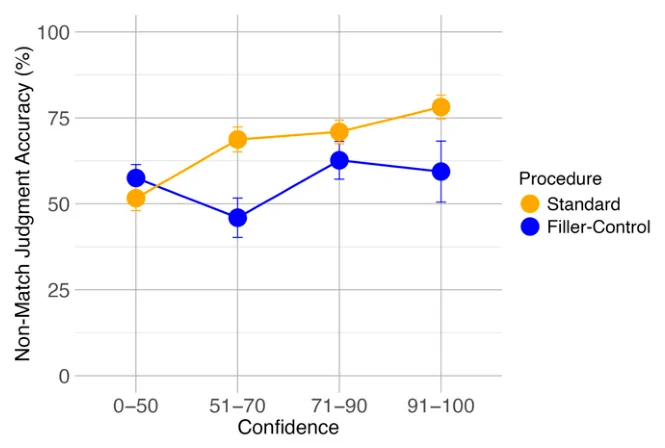
Experiment 1 Calibration Curves for Non-Match Judgments in the Standard and Filler-Control Procedures. Note. Error bars represent standard errors.

**Figure 3:**
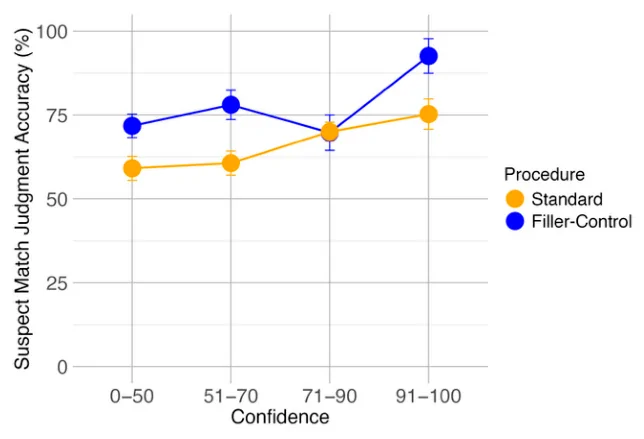
Experiment 1 Confidence–Accuracy Characteristic Curves Across Procedures. Note. Error bars represent standard errors.

**Figure 4:**
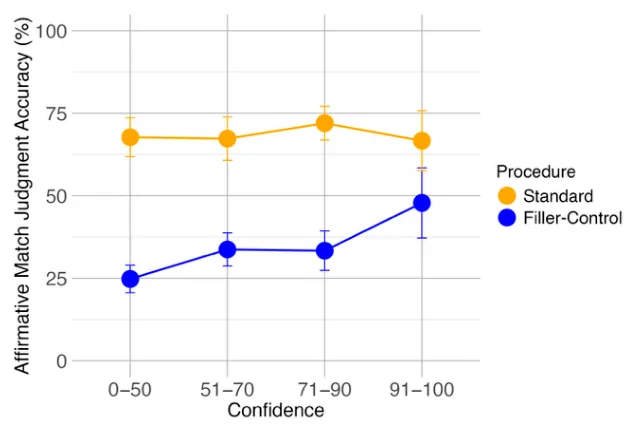
Experiment 2 Calibration Curves for Affirmative Match Judgments in the Standard and Filler-Control Procedures. Note. Error bars represent standard errors.

**Figure 5:**
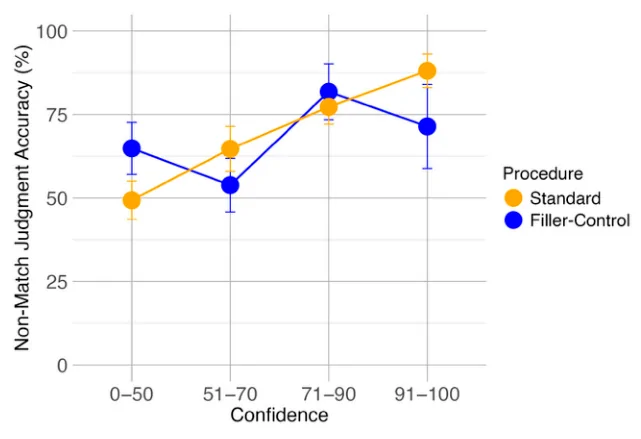
Experiment 2 Calibration Curves for Non-Match Judgments in the Standard and Filler-Control Procedures. Note. Error bars represent standard errors.

**Figure 6:**
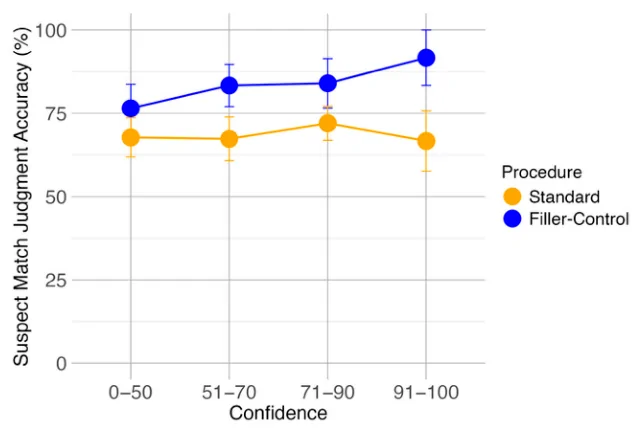
Experiment 2 Confidence–Accuracy Characteristic Curves in the Standard and Filler-Control Procedures. Note. Error bars represent standard errors.

**Figure S2:**
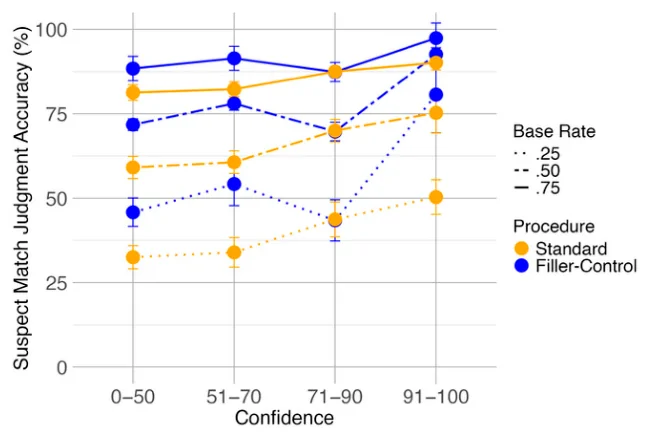
Experiment 1 Confidence-Accuracy Characteristic Curves in the Standard and Filler-Control Procedures at Varying Base Rates of Match Presence.

**Figure S4:**
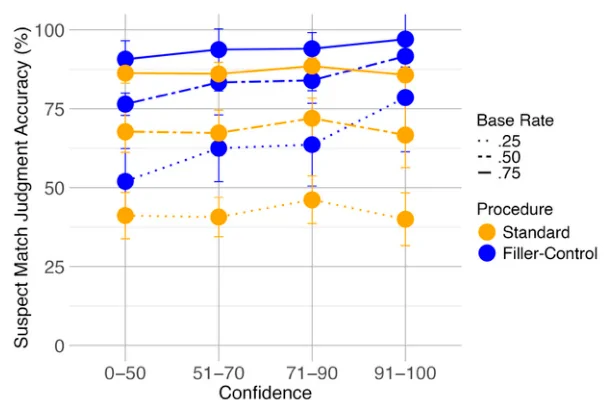
Experiment 2 Confidence-Accuracy Characteristic Curves in the Standard and Filler-control Procedures at Varying Base Rates of Match Presence.

**Table 2:** Experiment 1 confidence statistics for affirmative match judgments across procedures.

Procedure	Calibration	Over/Underconfidence	Resolution
Standard	0.02 *	0.006 *	0.01
Filler-Control	0.12 *	0.28 *	0.007

Note. * *p* < 0.001; indicates significant difference between procedures.

**Table 3:** Experiment 1 confidence statistics for non-match judgments across procedures.

Procedure	Calibration	Over/Underconfidence	Resolution
Standard	0.02 *	0.0007	0.04
Filler-Control	0.05 *	−0.007	0.005

Note. * *p* < 0.01; indicates significant difference between procedures.

**Table 6:** Experiment 2 confidence statistics for affirmative match judgments across procedures.

Procedure	Calibration	Over/Underconfidence	Resolution
Standard	0.05 *	−0.05 **	−0.01
Filler-Control	0.10 *	0.28 **	0.009

Note. * *p* < 0.05, ** *p* < 0.001; indicates significant difference between procedures.

**Table 7:** Experiment 2 confidence statistics for non-match judgments across procedures.

Procedure	Calibration	Over/Underconfidence	Resolution
Standard	0.01 *	−0.03	0.08
Filler-Control	0.05 *	−0.06	0.02

Note. * *p* < 0.05; indicates significant difference between procedures.
