# Supplementary material for: Does the Forensic Filler-Control Method Reduce Examiner Overconfidence? An Experimental Investigation Using Mock Fingerprint Examiners
Source: Behav Sci (Basel). 2025 Aug 31;15(9):1191. doi: 10.3390/bs15091191 (PMC12466420; doi:10.3390/bs15091191)
Supplement: Supplementary file 1 [file behavsci-15-01191-s001.zip › behavsci-3696796-supplementary.pdf]

Supplemental Materials

Experiment 1 Individual Filler-Match Rates

Table S1 displays the breakdown of individual filler-match rates for each evidence lineup. Suspect-match and non-match rates are included for reference.

Table S1

Experiment 1 Breakdown of Individual Filler-Match, Suspect-Match, and Non-Match Rates

Evidence Lineup	Suspect	Filler 1	Filler 2	Filler 3	Non-Match
Matching Evidence Lineup 1	52.27%	25.00%	1.14%	6.82%	14.77%
Matching Evidence Lineup 2	29.76%	11.90%	47.62%	2.38%	8.33%
Matching Evidence Lineup 3	87.64%	0.00%	2.25%	2.25%	7.87%
Matching Evidence Lineup 4	46.15%	17.58%	4.40%	13.19%	18.68%
Matching Evidence Lineup 5	34.83%	16.85%	21.35%	3.37%	23.60%
Matching Evidence Lineup 6	29.76%	9.52%	9.52%	29.76%	21.43%
Matching Evidence Lineup 7	32.22%	3.33%	24.44%	3.33%	36.67%
Matching Evidence Lineup 8	20.22%	11.24%	19.10%	15.73%	33.71%
Non-Matching Evidence Lineup 1	1.13%	72.73%	5.68%	0.00%	20.45%
Non-Matching Evidence Lineup 2	15.22%	12.00%	25.00%	29.35%	18.48%
Non-Matching Evidence Lineup 3	39.08%	14.94%	12.64%	13.79%	19.54%
Non-Matching Evidence Lineup 4	2.35%	8.24%	52.94%	14.12%	22.35%
Non-Matching Evidence Lineup 5	10.34%	19.54%	10.34%	35.63%	24.14%
Non-Matching Evidence Lineup 6	13.04%	7.61%	7.61%	37.00%	34.78%
Non-Matching Evidence Lineup 7	0.00%	9.30%	45.35%	9.30%	36.05%
Non-Matching Evidence Lineup 8	24.14%	0.00%	8.05%	29.89%	37.93%

Experiment 1 Receiver Operating Characteristic Analysis

To examine the overall potential for the two procedures to discriminate between matching and non-matching fingerprint sets, we used Receiver Operating Characteristic (ROC) analysis to compare investigator discriminability of the two procedures (Smith, Yang, & Wells, 2020; Smith & Neal, 2021; Yang & Smith, 2023). Investigator discriminability refers to how well the investigator (often the detective tasked with investigating the crime) can discriminate

between guilty vs. innocent suspects on the basis of the forensic analysis outcome (Smith, Yang, & Wells, 2020). To properly assess investigator discriminability in procedures involving fillers, one must use all three decision outcomes (suspect-match, filler-match, or non-match), as each provides diagnostic information about the suspect's likelihood of guilt. Accordingly, we generated full receiver-operating curves for each procedure using the *pROC* R package (Robin et al., 2011). The area under the curve reveals how well the procedure discriminates between matching and non-matching fingerprint sets.

Full ROC curves plot true- and false-positive rates across different investigator decision criteria. For example, the first operating point on an ROC curve represents the true and false positive rate under the most conservative criterion—when investigators judge the suspect as guilty based only on incriminating forensic judgments made with the highest level of confidence. The second operating point relaxes the decision criterion slightly—it represents the true and false positive rate when investigators judge the suspect as guilty based on incriminating forensic judgments made with either the highest or second-highest confidence level. The final operating point on the curve represents the true and false positive rate under the most liberal criterion—when investigators judge the suspect as guilty based on any forensic judgment, made with any level of confidence.

To construct the ROC curves, we ordered the operating points in terms of how strongly they support the conclusion that the suspect guilty (i.e., is the source of the crime-scene evidence). For the standard procedure, we ordered suspect-match judgments from highest to lowest confidence followed by non-match judgments from lowest to highest confidence (Smith & Neal, 2021). For the filler-control procedure, we ordered suspect-match judgments from highest to lowest confidence, followed by an operating point with all filler-match judgments

collapsed across confidence levels, and then non-match judgments from lowest to highest confidence (Ayala et al., 2022; Smith & Ayala, 2021; Smith et al., 2023). Thus, the curves plot cumulative true and false positive rates in order of stronger to weaker evidence of guilt and then from weaker to stronger evidence of innocence. The area under the curve (AUC) ranges from 0–1, with lower values indicating worse investigator discriminability and higher values indicating better investigator discriminability. However, because an AUC value of 0.50 indicates chance performance, the observed range of AUC values typically varies between 0.50 and 1.00.

As shown in Figure S1, the standard procedure ($AUC = .70$) resulted in significantly better investigator discriminability than did the filler-control procedure ($AUC = .62$, $p < .001$). Note, however, that the two curves intersect, indicating that neither procedure is objectively superior to the other. Instead, the filler-control method and standard method have different strengths. In the far left of the ROC space, which is comprised of suspect-match judgments, the curve for the filler-control method dominates the curve for the standard procedure. Thus, consistent with the PPV analyses reported in the manuscript, suspect-match judgments from the filler-control procedure are more diagnostic of guilt than are suspect-match judgments identifications from the standard procedure. Conversely, in the right side of the ROC space, which is comprised of filler-match judgments (in the filler-control procedure) and non-match judgments (in both procedures), the curve for the standard procedure dominates the curve for the filler-control method. This is again consistent with the findings reported in the manuscript: Exculpatory outcomes from the standard procedure are more diagnostic of innocence than are exculpatory outcomes from the filler-control procedure. More generally, when ROC curves cross over in this way, conclusions about which procedure is preferable depend on assumptions one

makes about the base rate of true matches and subjective preferences about the costs of different errors (Smith, Yang, & Wells, 2020).

Figure S1

Experiment 1 Investigator Full ROCs for the Standard and Filler-Control Procedures

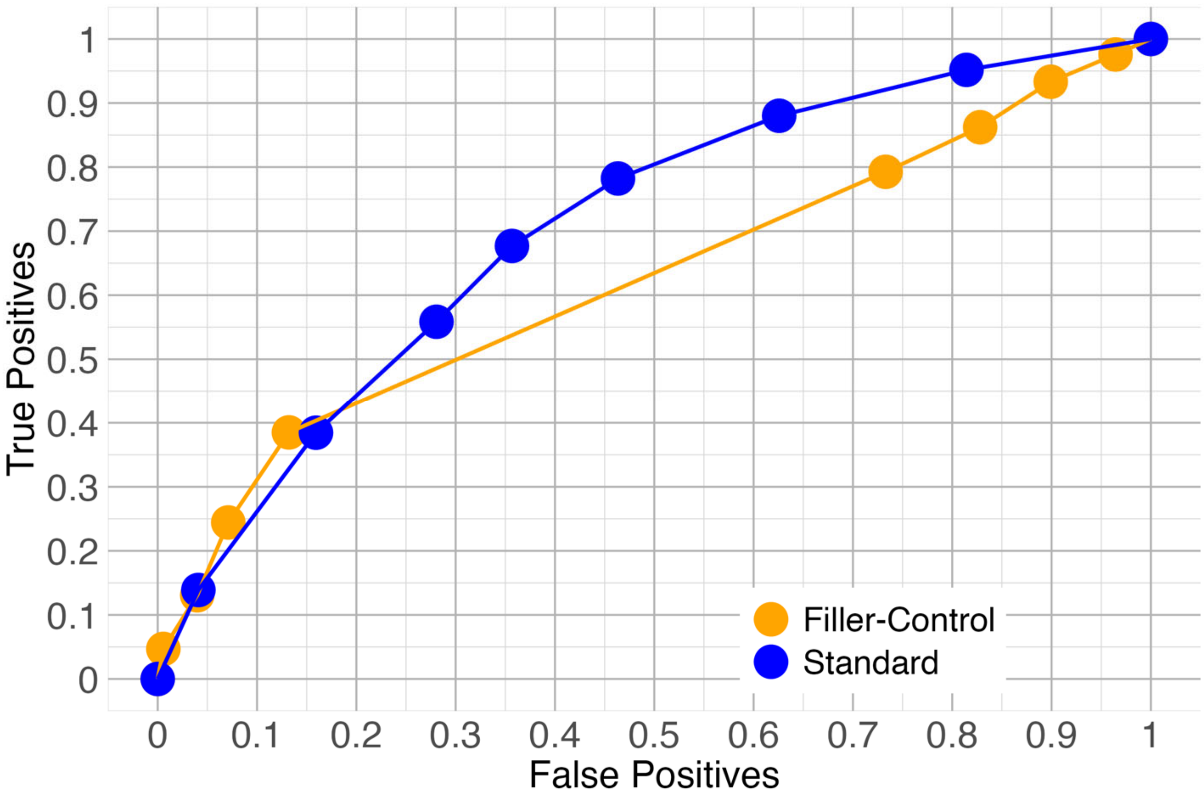

Experiment 1 Confidence-Accuracy Characteristic Analysis with Varying Base Rates of Match Presence

Figure S2 displays the confidence-accuracy characteristic curves for suspect-match judgments in the standard and filler-control procedures at varying base rates of match presence (.25, .50, .75). The filler-control procedure is associated with greater positive predictive value (PPV) than the standard procedure across these different base rates, but the difference between the two procedures decreases as the base rate increases.

Figure S2

Experiment 1 Confidence-Accuracy Characteristic Curves in the Standard and Filler-Control Procedures at Varying Base Rates of Match Presence

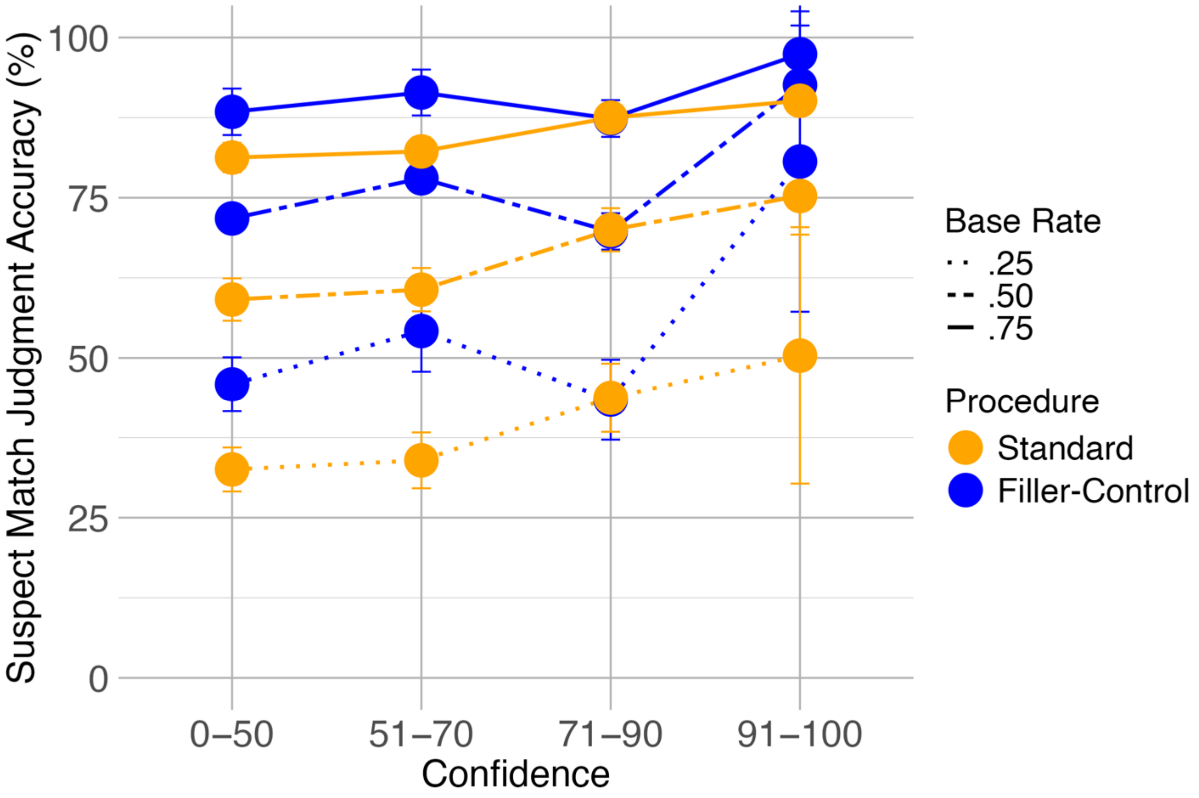

Note. Error bars represent standard errors.

Experiment 2 Individual Filler-Match Rates

Table S2 displays the breakdown of individual filler-match rates for each evidence lineup. Suspect-match and non-match rates are included for reference.

Table S2

Experiment 2 Breakdown of Individual Filler-Match, Suspect-Match, and Non-Match Rates

Evidence Lineup	Suspect	Filler 1	Filler 2	Filler 3	Non-Match
Matching Evidence Lineup 1	44.00%	28.00%	0.00%	8.00%	20.00%
Matching Evidence Lineup 2	33.33%	4.76%	33.33%	0.00%	28.57%
Matching Evidence Lineup 3	79.17%	0.00%	0.00%	12.50%	8.33%
Matching Evidence Lineup 4	60.71%	10.71%	0.00%	17.86%	10.71%

Matching Evidence Lineup 5	50.00%	20.83%	20.83%	0.00%	8.33%
Matching Evidence Lineup 6	30.77%	19.23%	19.23%	19.23%	11.54%
Matching Evidence Lineup 7	52.00%	4.00%	12.00%	0.00%	32.00%
Matching Evidence Lineup 8	21.74%	8.70%	17.39%	8.70%	43.48%
Non-Matching Evidence Lineup 1	4.17%	50.00%	12.50%	4.17%	29.17%
Non-Matching Evidence Lineup 2	14.29%	17.86%	17.86%	25.00%	25.00%
Non-Matching Evidence Lineup 3	24.00%	4.00%	20.00%	8.00%	44.00%
Non-Matching Evidence Lineup 4	0.00%	0.00%	38.10%	23.81%	38.10%
Non-Matching Evidence Lineup 5	12.00%	12.00%	12.00%	12.00%	52.00%
Non-Matching Evidence Lineup 6	8.70%	4.35%	8.70%	39.13%	39.13%
Non-Matching Evidence Lineup 7	0.00%	4.17%	54.17%	12.50%	29.17%
Non-Matching Evidence Lineup 8	11.54%	7.69%	7.69%	30.77%	42.31%

Experiment 2 Receiver Operating Characteristic Analysis

Figure S3 displays the receiver-operating curves for the standard procedure and filler-control procedure. We ordered the operating points using the same method described in Experiment 1. In contrast to the results from Experiment 1, there was no significant difference in investigator discriminability between the standard procedure ($AUC = .74$) and the filler-control procedure ($AUC = .69$, $p = .24$), though the trends were directionally consistent with the effects observed in Experiment 1. There was again a substantial crossover, with the filler control procedure showing better performance in the low left region of the ROC space and the standard procedure showing better performance in the upper right portion of the ROC space. This is consistent with patterns observed in the main manuscript and the ROC analyses reported above. For forensic science trainees, the forensic filler-control method again resulted in more reliable inculpatory evidence but less reliable exculpatory evidence compared to the standard procedure.

Figure S3

Experiment 2 Investigator Full ROCs for the Standard and Filler-Control Procedures

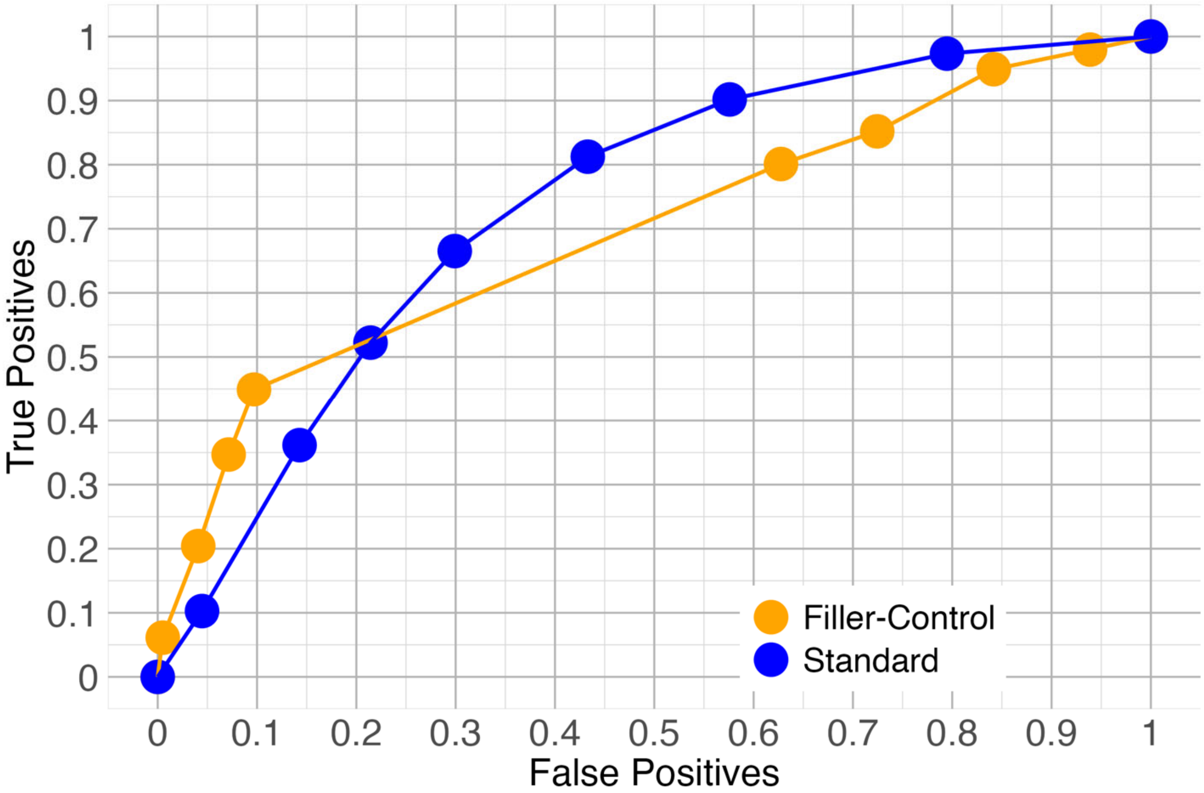

Note. Error bars represent standard errors.

Experiment 2 Confidence-Accuracy Characteristic Analysis with Varying Base Rates of Match Presence

Figure S4 displays the confidence-accuracy characteristic curves for suspect-match judgments in the standard and filler-control procedures at varying base rates of match presence (.25, .50, .75). Again, the filler-control procedure is associated with greater PPV than the standard procedure across these different base rates, but the difference between the two procedures decreases as the base rate increases.

Figure S4

Experiment 2 Confidence-Accuracy Characteristic Curves in the Standard and Filler-Control Procedures at Varying Base Rates of Match Presence

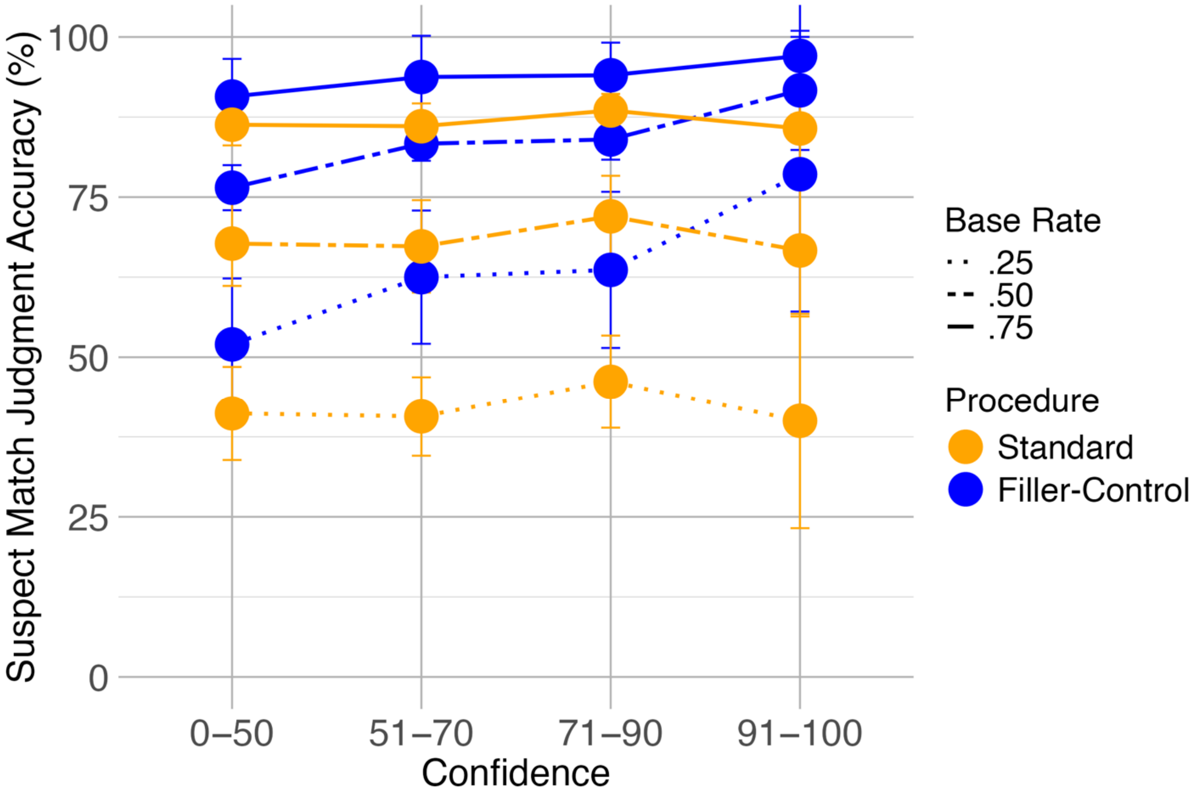

Note. Error bars represent standard errors.

References

Ayala, N. T., Smith, A. M., & Ying, R. C. (2022). The rule-out procedure: Increasing the potential for police investigators to detect suspect innocence from eyewitness lineup procedures. *Journal of Applied Research in Memory and Cognition, 11*(4), 489-499. https://doi.org/10.1037/mac0000018

Robin, X., Turck, N., Hainard, A., Tiberti, N., Lisacek, F., Sanchez, J-C., & Muller, M. (2011). pROC: an open-source package for R and S+ to analyze and compare ROC curves. *BMC Bioinformatics, 12*(77). https://doi.org/10.1186/1471-2105-12-77

Smith, A. M., & Ayala, N. T. (2021). Do traditional lineups undermine the capacity for eyewitness memory to rule out innocent suspects? *Journal of Applied Research in Memory and Cognition, 10*(2), 215-220. https://doi.org/10.1016/j.jarmac.2021.03.003

Smith, A, M., Ayala, N. T., & Ying, R. C. (2023). The rule out procedure: A signal-detection-informed approach to the calibration of eyewitness identification evidence. *Psychology, Public Policy, and Law, 29*(1), 19-31. https://doi.org/10.1037 /law0000373

Smith, A. M., & Neal, T. M. S. (2021). The distinction between discriminability and reliability in forensic science. *Science & Justice, 61*(4), 319-331. https://doi.org/10.1016/j.scijus. 2021.04.002

Smith, A. M., Yang, Y., & Wells, G. L. (2020). Distinguishing between investigator discriminability and eyewitness discriminability: A method for creating full receiver operating characteristic curves of lineup identification performance. *Perspectives on Psychological Science, 15*(3), 589-607. https://doi.org/10.1177/1745691620902426

Yang, Y. & Smith, A. M. (2023). fullROC: An R package for generating and analyzing eyewitness-lineup ROC curves. *Behavior Research Methods, 55*(3), 1259-1274. https://doi.org/10.3758/s13428-022-01807-6
